# The CLIP-Domain Serine Protease Homolog SPCLIP1 Regulates Complement Recruitment to Microbial Surfaces in the Malaria Mosquito *Anopheles gambiae*

**DOI:** 10.1371/journal.ppat.1003623

**Published:** 2013-09-05

**Authors:** Michael Povelones, Lavanya Bhagavatula, Hassan Yassine, Lee Aun Tan, Leanna M. Upton, Mike A. Osta, George K. Christophides

**Affiliations:** 1 Department of Life Sciences, Imperial College London, London, United Kingdom; 2 Department of Biology, American University of Beirut, Beirut, Lebanon; Institut Pasteur, France

## Abstract

The complement C3-like protein TEP1 of the mosquito *Anopheles gambiae* is required for defense against malaria parasites and bacteria. Two forms of TEP1 are present in the mosquito hemolymph, the full-length TEP1-F and the proteolytically processed TEP1_cut_ that is part of a complex including the leucine-rich repeat proteins LRIM1 and APL1C. Here we show that the non-catalytic serine protease SPCLIP1 is a key regulator of the complement-like pathway. SPCLIP1 is required for accumulation of TEP1 on microbial surfaces, a reaction that leads to lysis of malaria parasites or triggers activation of a cascade culminating with melanization of malaria parasites and bacteria. We also demonstrate that the two forms of TEP1 have distinct roles in the complement-like pathway and provide the first evidence for a complement convertase-like cascade in insects analogous to that in vertebrates. Our findings establish that core principles of complement activation are conserved throughout the evolution of animals.

## Author Summary

Mosquitoes are vectors of numerous human diseases including malaria. Disease transmission requires that microbes overcome the robust mosquito immune system. In the African malaria mosquito, the TEP1 protein that is homologous to mammalian complement factor C3 is shown to play a central role in mosquito immunity to malaria parasites and bacteria. In this study, we report that another mosquito protein belonging to a class of non-catalytic enzymes that are specific to arthropods is a core component of the mosquito complement-like immune pathway. We found that this new protein, named SPCLIP1, regulates the accumulation of TEP1 on malaria parasites and bacteria, and show that this can lead to distinct defense reactions including lysis and melanization of the pathogen. This work is valuable because it reveals novel insight into the regulation of mosquito complement on microbial surfaces such as those of the malaria parasites. Unraveling the molecular mechanisms regulating these defense responses may ultimately lead to the design of novel disease blocking strategies in the vector.

## Introduction

The mosquito *Anopheles gambiae* is the main vector of *Plasmodium falciparum* malaria in sub-Saharan Africa and hence directly responsible for the death of hundreds of thousands of people every year and for a devastating socioeconomic burden especially in endemic countries. Mosquitoes launch a potent immune attack leading to the killing of the majority of invading *Plasmodium* parasites. Multiple mechanisms are thought to participate in these anti-*Plasmodium* reactions, amongst them a latent pathway resembling vertebrate complement [1]. RNAi knockdown (kd) studies, based on the injection of double stranded RNA (dsRNA) into adult *A. gambiae* mosquitoes, have revealed important roles of components of the complement-like pathway in defense against the murine malaria parasite *Plasmodium berghei* [2]–[6]. There is also significant evidence for a role of this pathway in defense against the human parasite, *P. falciparum*, in laboratory infections of *A. gambiae* [3], [7]–[9].

Recent studies with natural *A. gambiae* populations revealed that the gene encoding the C3-like protein TEP1, a key player of the complement-like pathway, and the genomic locus encoding its interacting partner APL1C are under strong directional selection in an M form population but subject to balancing selection in another S form population [10], [11]. Despite the fact that distinct TEP1 alleles have been associated with resistance to *Plasmodium* parasites [2], [8], [11]–[13], the selective pressure on TEP1 is hypothesized to be driven by pathogens in larval habitats rather than those encountered by adults. This is further supported by the rather generic immune specificity of TEP1 that functions also in anti-bacterial [3], [14] and anti-fungal defense [15]. The polymorphic nature of TEP1 also suggests that the different alleles might follow different kinetics in interacting with LRIM1/APL1C as well as other TEP1 regulatory proteins, which could influence the efficiency of parasite killing or microbial clearance. Therefore, a better understanding of the mechanisms regulating complement activation and identification of the proteins involved will permit deciphering the functional relevance to *Plasmodium* of allelic interactions within this immune module on resistance.

The hallmark of activation of the mosquito complement-like pathway is the binding of TEP1 to microbial surfaces through a thioester bond, a reaction that is tightly linked to microbial killing [14]. TEP1 circulates in the mosquito hemolymph in two forms: the full-length form TEP1-F and the proteolytically processed form TEP1_cut_, corresponding to pro-C3 and the mature C3 protein after processing in the ER, respectively [14], [16]. Unlike C3, TEP1 lacks an anaphylatoxin domain and the exposed thioester bond of TEP1_cut_ is unstable [17]. TEP1_cut_ is stabilized in the hemolymph through interactions with a heterodimer of the leucine-rich repeat (LRR) proteins LRIM1 and APL1C, which seems to confer specificity upon TEP1 activity [5], [16]. While the structure and function of TEP1 and its C3 homolog are largely conserved from insects to mammals, LRIM1 and APL1C are thought to be specific to mosquitoes [18] raising interesting questions about the degree of structural and/or functional conservation between other modules of the complement pathway such as those that stabilize or amplify complement on microbial surfaces. The research presented here aimed to address these questions and provide novel mechanistic insights into the activation of the mosquito complement pathway during infection.

## Results

### SPCLIP1 is a component of the complement-like pathway

To identify novel components of the mosquito complement pathway, we searched for genes that exhibited significant co-regulation with *LRIM1* in a developmental transcriptome dataset of Expressed Sequence Tags (ESTs; [19]). Pearson correlation coefficient (PCC) identified 4 EST clusters showing similarity to *LRIM1* developmental expression greater than 0.95. Importantly, 3 of the 4 clusters were found to encode proteins that had been previously shown to physically interact with LRIM1, including APL1C (PCC 0.964), TEP1 (PCC = 0.978) and TEP4 (PCC = 0.965) [5], [16], [20]. The fourth EST cluster (PCC = 0.980) did not correspond to any gene model in the *A. gambiae* genome. It encodes a protein with CLIP and serine protease domains, previously identified as SPCLIP1 and shown to be involved in defense against *P. falciparum*, *P. berghei*, *Escherichia coli* and *Staphylococcus aureus* [3]. *SPCLIP1* maps within a genomic region encompassing 12 additional genes encoding proteins with CLIP and serine protease domains (Figure S1A). All residues corresponding to the serine protease catalytic triad (Asp-His-Ser) are substituted in SPCLIP1 indicating that it is non-catalytic (Figure S1B). Phylogenetic analysis places SPCLIP1 in the highly divergent CLIPE subfamily of non-catalytic CLIP-domain serine protease homologs (SPHs; Figure S1C).

**Figure 1:**
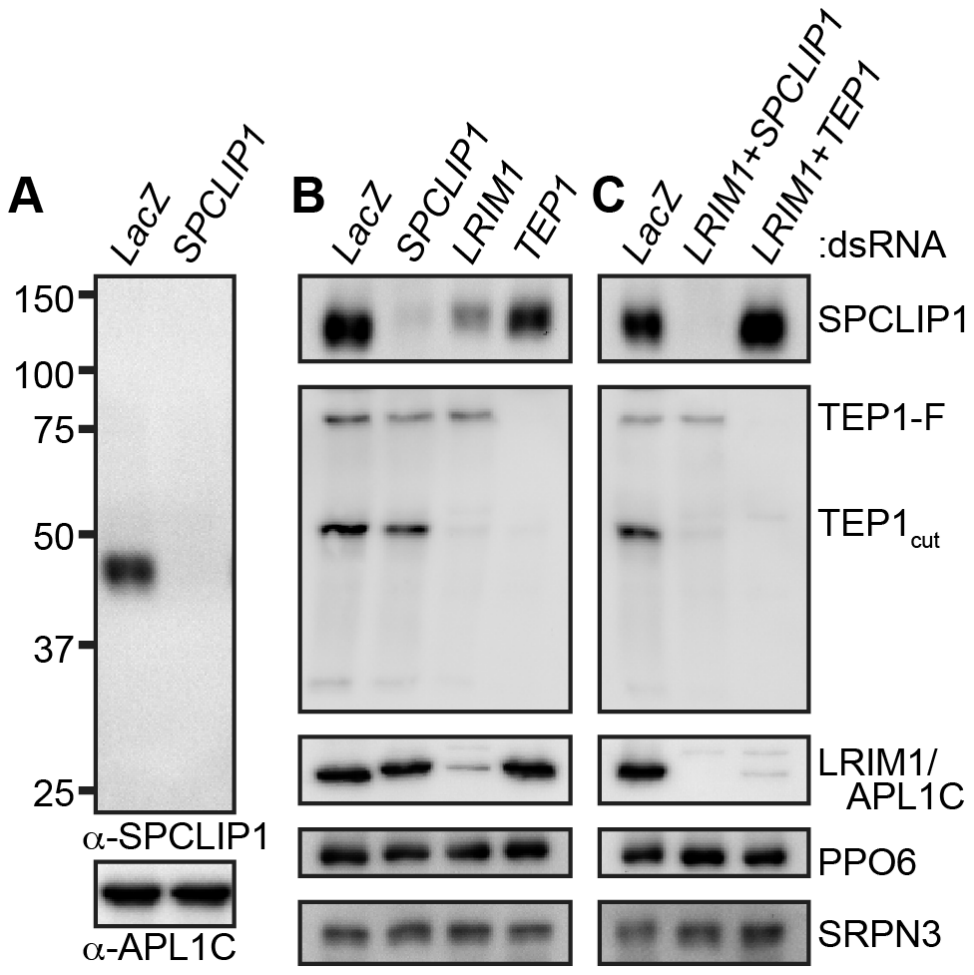
SPCLIP1 is a component of the mosquito complement cascade. (**A**) Western analysis of mosquito hemolymph collected 4 days after injection with *LacZ* or *SPCLIP1* dsRNA. The blot was initially probed with a polyclonal antibody raised against recombinant SPCLIP1 (top panel) and re-probed with an APL1C antibody (bottom panel) to confirm equal loading. (**B**)(**C**) Mosquito hemolymph collected 4 days after injection of *LacZ* dsRNA or silencing *SPCLIP1*, *LRIM1* or *TEP1* (or combinations of those) was analyzed by western blot using SPCLIP1, APL1C and TEP1 antibodies. Blots were re-probed with SRPN3 and PPO6 antibodies to confirm equal loading. The labels on the right indicate protein or complex detected. Images are representative of three independent biological replicates.

Co-regulation with LRIM1 and the previously reported knockdown phenotypes [3] were suggestive of SPLCLIP1 involvement in the *A. gambiae* complement-like pathway. To characterize SPCLIP1, we raised a polyclonal antibody against the entire protein and used it in western blots of adult mosquito hemolymph separated by non-reducing SDS-PAGE. The results showed that SPCLIP1 migrates at approximately 45 kDa, near its predicted 42 kDa molecular weight (Figure 1A). We examined whether the steady state levels of SPCLIP1 in the hemolymph are affected by silencing *LRIM1* or *TEP1*. While *TEP1* kd had no effect on SPCLIP1 levels, these were markedly reduced in *LRIM1* kd (Figure 1B). This decrease of SPCLIP1 parallels the near complete loss of TEP1_cut_ from the hemolymph of *LRIM1* or *APL1C* kd mosquitoes due to its accumulation on self-tissues (Figure 1B) [5], [16]. To determine if the reduction of SPCLIP1 in *LRIM1* kd is dependent on TEP1, we silenced *LRIM1* and *TEP1* simultaneously. Under these conditions, SPCLIP1 was restored to its baseline levels (Figure 1C). In contrast, silencing *LRIM1* and *SPCLIP1* together did not restore TEP1_cut_ levels, suggesting that SPCLIP1 functions downstream of TEP1_cut_, and that in *LRIM1* kd mosquitoes SPCLIP1 is likely to be sequestered on self-tissues together with TEP1_cut_.

### SPCLIP1 and TEP1 localization to ookinetes is mutually dependent

**Figure 2:**
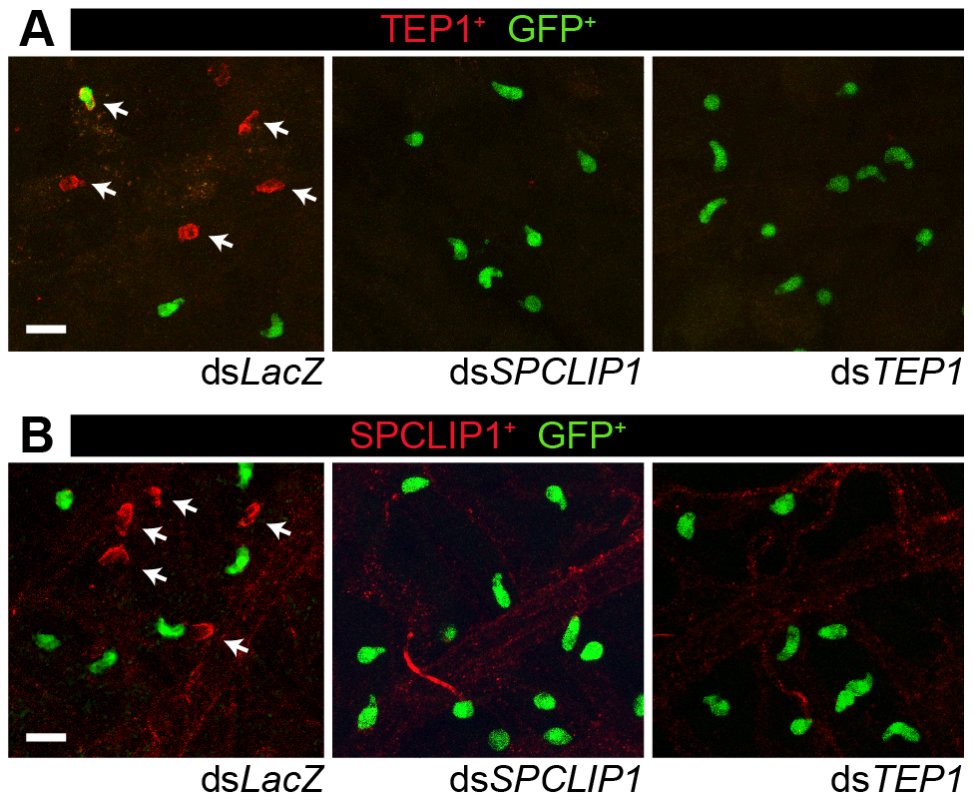
TEP1 and SPCLIP1 localization on dead parasites is mutually dependent. (**A**) TEP1 immunolocalization on the surface of GFP-expressing *P. berghei* parasites invading the mosquito midgut 26 h after infection. TEP1 positive parasites (arrows) do not express GFP and appear fragmented indicating that they are killed, while TEP1 negative parasites express GFP and are considered live. There is a dramatic reduction in TEP1 signal in mosquitoes treated with ds*SPCLIP1*. Lack of TEP1 signal in ds*TEP1* treated mosquitoes confirms the specificity of the antibody. A rare TEP1, GFP double positive parasite is visible in the upper left panel of the ds*LacZ* control. (**B**) SPCLIP1 immunolocalization on the surface of GFP-expressing *P. berghei* parasites invading the mosquito midgut epithelium 26 h after infection. SPCLIP1 positive parasites (arrows) are fragmented and lack GFP signal indicating they are dead. There is a dramatic reduction in SPCLIP1 signal in mosquitoes treated with ds*TEP1*. Lack of SPCLIP1 signal in the ds*SPCLIP1* treated mosquitoes confirms the specificity of the antibody. The background staining observed in all panels is non-specific antibody trapping by the trachea and muscle fibers present on the basolateral surface of the mosquito midgut. For both TEP1 and SPCLIP1 immunolocalization assays two independent biological replicates were performed with 5–10 midguts for each dsRNA. Panels are representative confocal projections of an approximately 20 µm thick section of the midgut basolateral surface. The scale bar is 10 µm.

We investigated the role of SPCLIP1 in TEP1 binding to *P. berghei*. It has been previously established that TEP1 binds to the surface of *P. berghei* ookinetes as they traverse the mosquito midgut epithelium and come into contact with the hemolymph [2]. In *SPCLIP1* kd mosquitoes, TEP1 staining on the ookinete surface was inhibited (Figure 2A). This, together with the TEP1-dependent reduction of SPCLIP1 from the hemolymph following *LRIM1* kd, led us to hypothesize that SPCLIP1 is recruited to the parasite surface during infection. To test this, SPCLIP1 was immunolocalized in midgut epithelium 26 h after infection. We observed robust SPCLIP1 signal on dead ookinetes, judged by the loss of their cytoplasmic GFP signal (Figure 2B). Given that TEP1 is also highly prevalent on dead ookinetes, this result indicates that SPCLIP1 and TEP1 likely co-localize to the same ookinetes; however, we could not simultaneously assay their distribution since both antibodies were raised in the same host species. No SPCLIP1 staining was observed in midgut epithelia dissected from *SPCLIP1* kd mosquitoes, showing that the antibody is specific. Importantly, SPCLIP1 staining on the ookinete surface was inhibited after *TEP1* kd. This suggests that the localization of TEP1 and SPCLIP1 to ookinetes is mutually dependent (Figure 2B).

### SPCLIP1 is required for the utilization of TEP1-F

**Figure 3:**
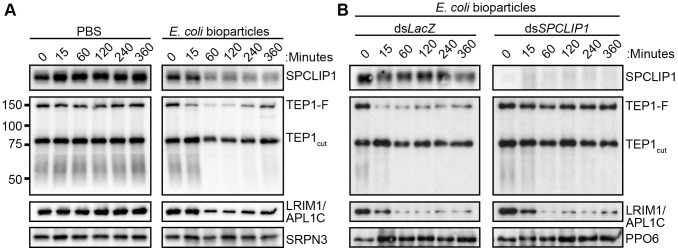
SPCLIP1 is required for the utilization of TEP1-F. (**A**) Western blot analysis of hemolymph collected from mosquitoes after injection with PBS (left) or *E. coli* bioparticles (right) using a panel of different antibodies. Full-length and processed TEP1 are indicated as TEP1-F and TEP1_cut_, respectively. Re-probing with SRPN3 was used to confirm equal loading. (**B**) Western blot analysis of hemolymph collected from control *LacZ* dsRNA-injected (left) and *SPCLIP1* kd (right) mosquitoes after injection with *E. coli* bioparticles. Re-probing with PPO6 was used to confirm equal loading. Images are representative of three independent biological replicates.

TEP1 present on microbial surfaces during infection may originate either from the TEP1_cut_ or the TEP1-F pools. To clarify this point and investigate further the functional relationship between the two forms of TEP1 and SPCLIP1, we developed an alternative infection model that allowed us to monitor temporally and quantitatively the dynamics of the examined proteins after injection of *E. coli* bioparticles (chemically killed bacteria) into the hemocoel. This infection model offers the advantage of tight temporal monitoring of rapid immune responses such as those of complement, which occur within minutes of microbial exposure to the hemolymph. Hemolymph was collected from groups of mosquitoes at 15, 60, 120, 240 and 360 minutes post injection with bacteria or PBS (i.e. control) and proteins were analyzed by western blot. The results showed strong reduction in SPCLIP1, the LRIM1/APL1C complex, and TEP1-F levels in mosquito hemolymph after injection of *E. coli* bioparticles (Figure 3A). A marked reduction of these proteins was already observed at 60 min after injection and persisted up to 240 min when LRIM1/APL1C and TEP1-F levels began to rise. The kinetics of TEP1-F reduction demonstrate that this form of TEP1 is consumed quickly in the immune response to infection, in contrast to TEP1_cut_, which does not seem to vary significantly during that process, at least within the examined timeframe. In addition to the well-defined TEP1-F and TEP1_cut_ bands, we also observed a broadly stained TEP1-specific smear at 50–60 kDa exhibiting depletion kinetics following bioparticle challenge similar to that of TEP1-F (Figure 3A). These C-terminal TEP1 fragments have been previously described [14]; whether they represent functional forms of TEP1 or are products of TEP1-F turnover remains to be determined.

LRIM1/APL1C and SPCLIP1 exhibited similar depletion kinetics as TEP1-F following bioparticle injections (Figure 3A), suggesting that these proteins are either required for TEP1-F utilization or are independently consumed in the immune reactions. To address this, we monitored the effect of *SPCLIP1* silencing on the infection-dependent depletion of TEP1-F. Western blot analysis of hemolymph collected from *SPCLIP1* and control *LacZ* kd mosquitoes challenged with *E. coli* bioparticles demonstrated that the loss of TEP1-F is abolished in *SPCLIP1* kd mosquitoes compared to controls (Figure 3B), indicating that SPCLIP1 acts upstream of TEP1-F and is indeed required for the infection-induced loss of this protein. In contrast, the depletion of LRIM1/APL1C was not restored in the hemolymph of *SPCLIP1* kd mosquitoes. Together, these data suggest that activation of mosquito complement by the LRIM1/APL1C/TEP1_cut_ complex is a separate event upstream of the SPCLIP1-dependent complement amplification process that is poised to transform initial pathogen recognition into a robust attack.

### SPCLIP1 is required for TEP1-F to TEP1_cut_ conversion on microbial surfaces

**Figure 4:**
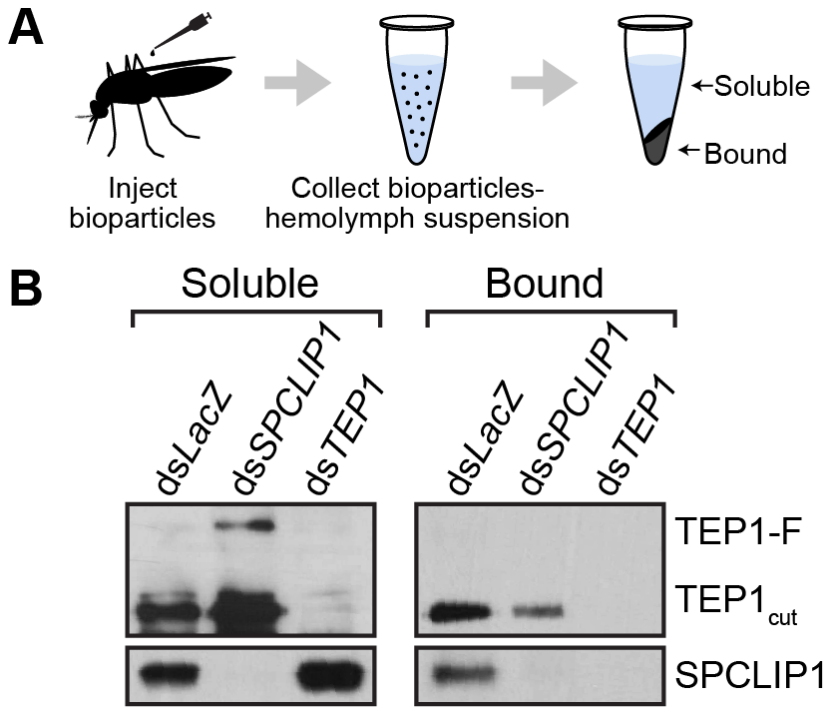
SPCLIP1 and TEP1_cut_ are localized on the surface of *E.* *coli* bioparticles. (**A**) Schematic overview of sample preparation. Hemolymph containing *E. coli* bioparticles was recovered 15 min after injection into mosquitoes after gene silencing. The bacteria were separated by centrifugation and the soluble fraction was collected. The bacterial pellet was washed with buffer and extracted for analysis. (**B**) Western blot analysis of soluble and bioparticle bound fractions using antibodies against TEP1 and SPCLIP1. Images are representative of two independent biological replicates.

An important aspect of the complement system is its specific activation on microbial surfaces. In order to address whether the observed reduction in SPCLIP1 and TEP1-F levels in the hemolymph after injection of *E. coli* bioparticles is due to their sequestration on bioparticle surfaces, we designed an assay that allows quantitative assessment of *E. coli*-bound versus hemolymph soluble pools of these proteins. *E. coli* bioparticles were injected into mosquito hemocoel, and hemolymph was extracted 15 min after injection. Bioparticles were separated from the hemolymph by centrifugation, washed extensively and their surface-bound proteins eluted for western blot analysis (Figure 4A). The results showed that SPCLIP1 was present in the *E. coli*-bound fraction in ds*LacZ* control mosquitoes (Figure 4B), which explains its reduced levels in the hemolymph after bacterial challenge and is consistent with its localization to ookinetes. In *TEP1* kd mosquitoes, SPCLIP1 was lost from the *E. coli*-bound fraction and became enriched in the soluble fraction, indicating that TEP1 is required for SPCLIP1 recruitment to bacterial surfaces.

This assay also allowed us to monitor which of the two forms of TEP1 associates with the bacterial surface. In ds*LacZ* treated mosquitoes, TEP1-F was not detected in the *E. coli*-bound fraction, despite being almost fully depleted from the soluble material, in contrast to TEP1_cut_, which was clearly present. These data are consistent with those reported previously using a cell culture assay and showing that bacteria only bound TEP1_cut_ when incubated with the conditioned medium of a hemocyte-like cell line that contained both forms of TEP1 [14]. Importantly, TEP1_cut_ signal in the bound material was dramatically reduced by *SPCLIP1* kd, concomitant with the detection of TEP1-F in the soluble fraction. These data indicate that TEP1_cut_ accumulating on the surface of *E. coli* is generated from TEP1-F and that its conversion requires recruitment of SPCLIP1 and a yet unidentified protease to the bacterial surface.

### Microbial infection promotes SPCLIP1 interaction with TEP1

**Figure 5:**
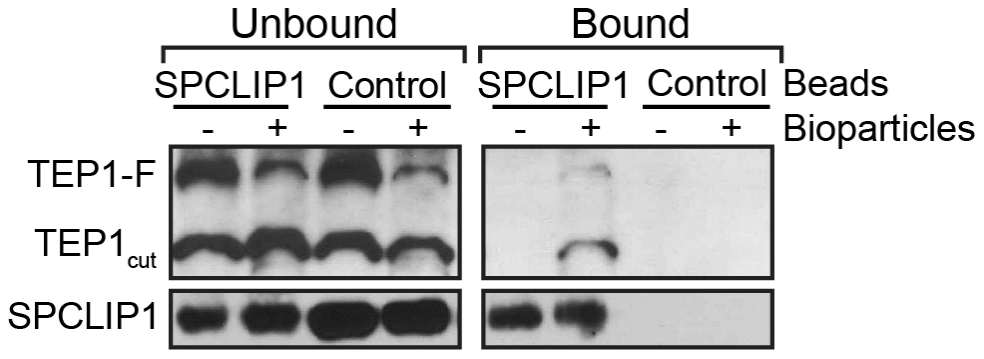
SPCLIP1 and TEP1 interact after challenge with *E.* *coli* bioparticles. IP beads containing SPCLIP1 antibody or control beads were used to capture proteins from hemolymph 15 min after injection with *E. coli* bioparticles (+) or PBS (−). The beads were separated and samples of the unbound and bound fractions were analyzed by western blot under reducing and non-reducing conditions for TEP1 and SPCLIP1, respectively. Images are representative of two independent biological replicates.

The functional association between SPCLIP1 and TEP1 including their cooperative recruitment to microbial surfaces suggested that these two proteins might physically interact. To examine this possibility, we performed an immunoprecipitation (IP) assay on hemolymph samples collected from mosquitoes following challenge with *E. coli* bioparticles using beads cross-linked to an affinity purified SPCLIP1 antibody. IP beads lacking antibody and mock bioparticle challenge (PBS injection) served as controls. The results revealed that SPCLIP1 was less abundant in the unbound fraction and significantly enriched in the bound fraction (Figure 5). In contrast, SPCLIP1 was not detected on control beads and the protein remained highly abundant in the unbound fraction. When samples were probed for TEP1, a signal for TEP1_cut_ and a faint but clear TEP1-F signal were observed in the SPCLIP1 IP bound fraction. These bands were detectable only in samples collected from bioparticle challenged mosquitoes. These data indicate that SPCLIP1 and TEP1 can interact and that this interaction is induced by infection. These data raise the possibility that these proteins interact first in the hemolymph prior to their localization on microbial surfaces. Alternatively, membrane-bound complexes containing TEP1 and SPCLIP1 may leach off the surface during sample preparation. Whether this interaction is direct or mediated by another factor remains to be determined.

### SPCLIP1 is required for activation of the melanization cascade

**Figure 6:**
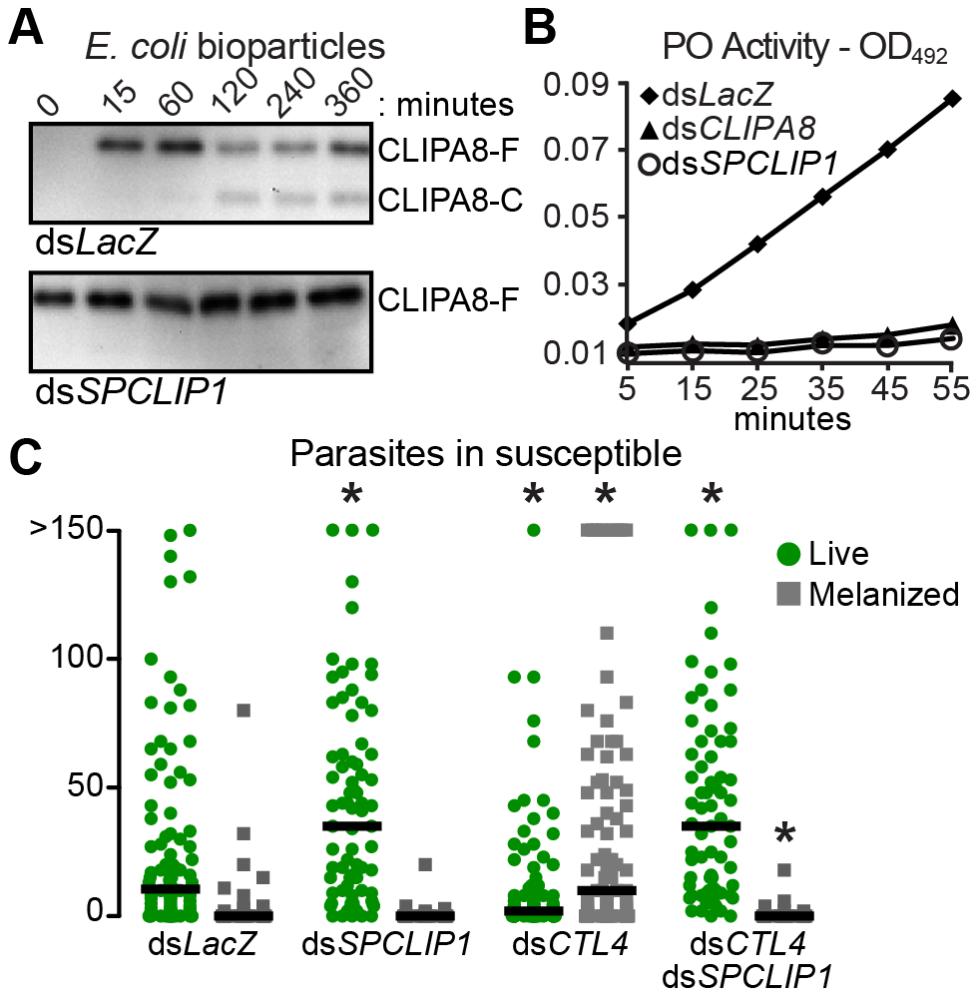
SPCLIP1 is required for triggering the melanization cascade. (**A**) Reducing western blot analysis of CLIPA8 in hemolymph collected from control *LacZ* dsRNA-injected (top) and *SPCLIP1* kd mosquitoes (bottom) after injection with *E. coli* bioparticles. Full-length and cleaved CLIPA8 are labeled CLIPA8-F and CLIPA8-C, respectively. Images are representative of two independent biological replicates. (**B**) PO activity measured in hemolymph samples collected from ds*LacZ*, ds*SPCLIP1* and ds*CLIPA8* treated mosquitoes 6 h after injection with bacteria. Data are representative of two independent biological replicates. See also Figure S2. (**C**) GFP-expressing *P. berghei* oocysts (green circles) and melanized ookinetes (gray squares) in ds*LacZ*, ds*SPCLIP1*, ds*CTL4* and ds*CTL4*/ds*SPCLIP1* injected mosquitoes 7 days post infection were counted. Lines indicate median infection intensity values. Data were combined from three independent biological replicates. For statistical analysis, ds*CTL4* and ds*SPCLIP1* injected mosquitoes were compared to ds*LacZ* while ds*CTL4*/ds*SPCLIP1* injected mosquitoes were compared to ds*CTL4*. Asterisks indicate Kruskal-Wallis P-values<0.01.

It has been previously shown that bacterial inoculation into the mosquito hemolymph leads to rapid activation cleavage of CLIPA8, a key SPH regulator of bacteria [21] fungi [15], and *Plasmodium* melanization [22]. We examined whether SPCLIP1 is required for CLIPA8 activation in the mosquito hemolymph following *E. coli* bioparticle injection. As shown in Figure 6A, silencing *SPCLIP1* inhibited completely CLIPA8 cleavage, suggesting that SPCLIP1 is required for activation of the melanization cascade.

The final steps of melanization are catalyzed by phenoloxidase (PO) which is secreted as a pro-enzyme (PPO) and activated by proteolytic cleavage in response to infection. We directly examined whether SPCLIP1 is essential for PPO activation by monitoring PO activity in the mosquito hemolymph after bacterial injection. Indeed, *SPCLIP1* kd resulted in a strong decrease in PO activity relative to ds*LacZ*-injected controls, which is comparable to that observed in *CLIPA8* kd mosquitoes (Figure 6B). Similar to *SPCLIP1* kd, silencing *TEP1* also resulted in strong inhibition of both CLIPA8 cleavage and PPO activation (Figure S2). These data demonstrate that activation of the melanization cascade is dependent on SPCLIP1-mediated TEP1 accumulation on the bacterial surface.

We next tested the function of SPCLIP1 in *P. berghei* melanization using as a model *CTL4* kd mosquitoes which melanize nearly all ookinetes soon after they traverse the mosquito midgut and before they develop into oocysts [4]. Indeed, silencing *CTL4* alone resulted in a marked decrease of the oocyst numbers and a reciprocal increase in melanized ookinetes, but concomitant silencing of *SPCLIP1* completely blocked ookinete melanization and led to an increase in oocysts comparable to that of *SPCLIP1* kd alone (Figure 6C). A similar inhibition of parasite melanization has been observed after silencing TEP1 or LRIM1/APL1C [2], [4], [5]. These data reveal that, as with bacterial melanization, SPCLIP1-mediated accumulation of TEP1 on the ookinete surface is required for parasite melanization.

## Discussion

**Figure 7:**
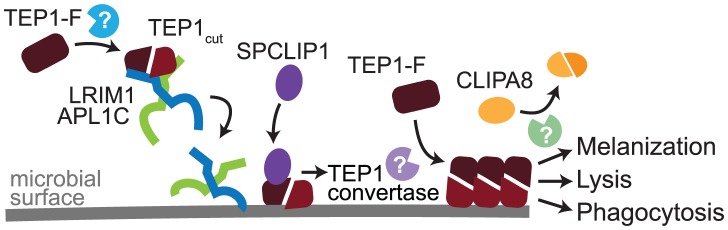
Model of TEP1 convertase formation. In steady state hemolymph a pool of TEP1-F is processed by an unknown protease to generate TEP1_cut_, which interacts and circulates with the LRIM1/APL1C complex. Recognition of microbial surfaces leads to deposition of LRIM1/APL1C and TEP1_cut_ and subsequent recruitment of SPCLIP1. An unknown catalytically active protease is then recruited generating the mature TEP1 convertase, which processes TEP1-F causing it to rapidly interact with nearby surfaces. Steady state processing of TEP1-F and that performed by the TEP1 convertase are distinct, as only the latter requires SPCLIP1. Formation of the TEP1 convertase is required for phagocytosis, lysis, or CLIPA8 cleavage by an unknown protease and subsequent activation of the melanization cascade.

Here we characterize SPCLIP1, a non-catalytic CLIP-domain serine protease of the malaria vector mosquito *A. gambiae*, which localizes to the surface of *P. berghei* ookinetes and *E. coli* promoting rapid accumulation of the complement C3-like protein TEP1. Our results demonstrate that SPCLIP1 regulates a complement convertase-like activity henceforth referred to as TEP1 convertase. The TEP1 convertase is functionally analogous to the vertebrate C3 convertase, the formation of which is triggered by binding of antibodies or innate pattern recognition proteins on the microbial surfaces, or by spontaneous activation of C3 following hydrolysis of its thioester. The trigger for the formation of the TEP1 convertase is thought to be the binding on the microbial surface of TEP1_cut_ which circulates in the mosquito hemolymph together with the LRIM1/APL1C complex (Figure 7). LRIM1 and APL1C possess LRR domains, a feature that is versatile in its binding properties and common in pattern recognition receptors involved in host defense in animals and plants [23]. Therefore, the LRIM1/APL1C complex may play a dual role in the mosquito complement-like pathway by stabilizing TEP1_cut_ in the hemolymph and delivering it to the microbial surface upon infection. Given that the LRIM1 and APL1C belong to a mosquito-specific family of LRR proteins [5] whereas TEPs are widely conserved [24], different triggers of complement activity are likely to exist in other insects. A number of different putative pattern recognition receptors have been identified to play a role in TEP1-dependent defense against bacteria and malaria parasites [4], [6], [25]–[27] raising the possibility that mosquitoes may also have multiple recognitions systems that can activate the TEP1 convertase. It has been proposed that nitration of malaria parasites during their passage through the mosquito midgut epithelium is required for TEP1 binding [28]. Whether microbe nitration can trigger recognition by LRIM1/APL1C or other putative recognition receptors remains to be determined.

A study using recombinant proteins and an allele of TEP1 from mosquitoes that are refractory to *Plasmodium* has shown that the LRIM1/APL1C complex binds TEP1_cut_ lacking an intact thioester, and that TEP1_cut_ precipitates out of solution in the absence of LRIM1/APL1C [17]. A more recent study using a TEP1_cut_ allele from susceptible mosquitoes has revealed that the LRIM1/APL1C complex can interact with TEP1_cut_ with an active thioester [29]. These *in vitro* studies have led the authors to speculate that a complex between LRIM1/APL1C and TEP1_cut_ may function *in vivo* as a TEP1 convertase. It remains unknown whether TEP1_cut_ lacks an intact thioester *in vivo*, and whether its localization on mosquito tissues in the absence of LRIM1/APL1C is the result of protein precipitation or autoimmune attack by an active thioester motif [5], [16]. The TEP1_cut_ dependent SPCLIP1 depletion favors the hypothesis of an autoimmune attack that is tightly regulated to prevent collateral damage to host tissues. Indeed, SPCLIP1 loss from the hemolymph following artificial induction of TEP1_cut_ attack of self-tissues is not accompanied by TEP1-F depletion, suggesting that downstream negative regulators prevent the full formation of the TEP1 convertase and/or that additional positive factors similar to vertebrate properdin may be required to stabilize the convertase on microbial surfaces.

SPCLIP1 lacks catalytic serine protease activity and likely acts as a regulatory component of the TEP1 convertase. Hence, an unidentified protease and possibly other factors are expected to also contribute to the mature convertase, catalyzing the activation cleavage of TEP1-F. The role of non-catalytic serine proteases as cofactors for active proteases is well documented in insects with examples from *Holotrichia diomphalia* [30], *Manduca sexta* [31] and *Drosophila melanogaster* [32].

The SPCLIP1-dependent rapid loss of TEP1-F from the hemolymph of bioparticle injected mosquitoes and the observation that *SPCLIP1* kd in naive mosquitoes does not alter TEP1-F levels, suggests that the TEP1_cut_ cargo circulating as a complex with LRIM1/APL1C is generated through a different mechanism than that produced by the TEP1 convertase. Of note, while bioparticle injection almost depletes TEP1-F from the hemolymph, only a minor reduction in TEP1_cut_ levels is observed most significantly at 60 min post injection. A plausible explanation for this observation is that TEP1-F is converted to TEP1_cut_ prior to binding the bacterial surface, a fraction of which remains soluble in the hemolymph throughout the timeframe of the experiment.

Regardless of the activation mechanism, the C3 and TEP1 convertases function in very similar ways to recruit additional C3 and TEP1, respectively, from precursor pools onto the microbial surface, and to initiate diverse effector cascades. In vertebrates, accumulation of the C3 cleavage product, C3b, on microbial surfaces triggers phagocytosis as well as assembly of the membrane attack complex that causes microbial lysis. In mosquitoes, in addition to triggering phagocytosis of bacteria [14], [33] and lysis of malaria parasites [2], [3], TEP1 accumulation on microbial surfaces triggers the PO cascade leading to melanization. Therefore, the strategy of complement driving diverse effector functions is ancient and not specifically co-opted by vertebrates. It remains to be further investigated whether this system is indeed an example of convergent evolution rooted to the functional conservation of thioester-containing proteins, a hypothesis consistent with our earlier findings that this pathway appears to have evolved *de novo* in each mosquito species by “bricolage” assemblages of the most suitable available components [34].

## Materials and Methods

### Ethics statement

This study was carried out in strict accordance with the United Kingdom Animals (Scientific Procedures) Act 1986. The protocols for maintenance of mosquitoes by blood feeding and for infection of mosquitoes with *P. berghei* by blood feeding on parasite-infected mice were approved and carried out under the UK Home Office License PLL70/7185 awarded in 2010. The procedures are of mild to moderate severity and the numbers of animals used are minimized by incorporation of the most economical protocols. Opportunities for reduction, refinement and replacement of animal experiments are constantly monitored and new protocols are implemented following approval by the Imperial College Ethical Review Committee.

### Mosquito maintenance, gene silencing and infection

*A. gambiae* G3 strain was maintained and assayed for infection with *P. berghei* CON_GFP_ strain as described previously [20]. Single and double knockdown experiments and parasite counts in dissected midguts were performed as described previously [5]. Primers used for synthesis of double stranded RNA have been reported elsewhere *LRIM1*, *TEP1*, *CTL4* [4], [35]; *SPCLIP1* [3].

### Generation and purification of SPCLIP1 antibody

The entire *SPCLIP1* open reading frame lacking the endogenous signal peptide and stop codon was cloned into the *pIEx10* insect cell expression plasmid (Novagen) incorporating a C-terminal 10× HIS-tag using the primers:

For: GACGACGACAAGATGAACTTCCCCGTTGGGAAATTTC

Rev: GAGGAGAAGCCCGGTTTATCGAAGCTGATCGGATCGGG

The underlined sequences are extensions to allow ligase-independent cloning [5]. Sf9 cells adapted for growth in serum-free medium (Invitrogen) stably secreting SPCLIP1^HIS^ were generated by selection with 1 mg/mL G418 following co-transfection using Escort IV (Sigma) of *pIEx10-SPCLIP1^HIS^* and *pIE1-neo* (Novagen). Clones of resistant cells were analyzed by western blot for the presence of SPCLIP1^HIS^ in their conditioned medium and the line with the highest expression was chosen for protein production. SPCLIP1^HIS^ was purified from 500 mL of conditioned medium using a 1 mL HisTrap column attached to an ÄKTA purifier (GE Healthcare). Bound protein was eluted in 15 mL of PBS containing 500 mM imidazole pH 8.0. Purified SPCLIP1^HIS^ was quantified by Bradford assay and by coomassie staining of SDS-PAGE gels. The purified protein was used to generate a rabbit polyclonal antibody (Eurogentec). SPCLIP1 antibody was affinity purified from the positive immune serum by passage over an AminoLink column (Pierce) containing covalently bound SPCLIP1^HIS^.

### Bioparticles challenge

A 20 mg/mL suspension of fluorescein or pHrodo labeled *E. coli* K-12 strain bacterial bioparticles (Invitrogen) in sterile PBS was injected into the mosquito hemocoel (∼4×10^5^ bacteria in 69 nL). Hemolymph was collected directly into non-reducing SDS-PAGE sample buffer from groups of 30–40 mosquitoes 15, 60, 120, 240 and 360 min after the challenge and analyzed by reducing and non-reducing western as described previously [5]. Bioparticles surface extraction was performed by collecting in protein LoBind tubes (Eppendorf) hemolymph from 60 mosquitoes into 60 µL of 15 mM Tris (pH 8.0) containing 1× protease inhibitor cocktail (complete EDTA free, Roche) 15 min after bacterial injection. The soluble (unbound) fraction was collected after pelleting the bacteria by centrifugation for 4 min at 6000 g at 4°C and then supplemented with SDS-PAGE buffer. The bacterial pellet was washed with 400 µL of 15 mM Tris (pH 8.0) and the bound fraction was extracted with 25 µL SDS-PAGE sample buffer. Western blot analysis was performed using 25 µL of each sample.

### Immunoprecipitation and western analysis

Western blot analysis for TEP1, LRIM1/APL1C, SRPN3, CLIPA8 and PPO6 was performed as previously described [5], [21]. The affinity purified rabbit α-SPCLIP1 antibody was used to probe western blots at a 1∶1000 dilution of antibody in PBS containing 0.05% Tween 20 and 3% milk for 1 h at room temperature using. Co-immunoprecipitation reactions were performed using the Pierce Co-IP kit according to the manufacturer's protocol (ThermoScientific). Hemolymph was collected from 100 mosquitoes into 200 µL ice-cold PBS containing 0.05% Triton X-100, supplemented with 1× protease inhibitor cocktail 15 min after PBS or *E. coli* bioparticle injection (69 nL of 4 mg/mL; ∼8×10^4^ particles). The samples were centrifuged at 4000 g for 5 min to remove mosquito and bacterial cells. 40 µL of a 1∶1 slurry of PBS and agarose beads containing crosslinked affinity purified α-SPCLIP1 antibody or control beads were added to the cleared hemolymph samples and mixed overnight at 4°C on a rotating wheel. The unbound fraction was collected and supplemented with SDS-PAGE buffer. Then the beads were washed five times with collection buffer and bound material was eluted two times with 100 µL of elution buffer (0.2% SDS and 0.1% Tween-20 in 50 mM Tris pH 8.0). The eluents were pooled and supplemented with SDS-PAGE buffer. Western blot analysis was performed by loading 40 µL of each sample. Reducing samples were made by addition of 2-mercaptoethanol to a final concentration of 2.5%.

### PPO activation and CLIPA8 cleavage

Cleavage of CLIPA8 was assayed in samples of hemolymph analyzed under reducing conditions as described previously [21]. PPO activation was determined assaying the conversion of L-DOPA to Dopachrome in samples of mosquito hemolymph collected after bacterial challenge [36].

### Immunolocalization of TEP1 and SPCLIP1

TEP1 and SPCLIP1 were immunolocalization to ookinetes 26 h after *P. berghei* infection. Mosquito midguts were prepared and analyzed as previously described [5]. The SPCLIP1 antibody was used at a 1∶250 dilution. Images were acquired on a Zeiss LSM 710 META confocal.

### VectorBase gene identifiers

LRIM1, AGAP006348; APL1C, AGAP007033; TEP1, AGAP010815; TEP4, AGAP010812; CLIPA1, AGAP011791; CLIPA2, AGAP011790; CLIPA4, AGAP011780; CLIPA5, AGAP011787; CLIPA6, AGAP011789; CLIPA7, AGAP011792; CLIPA8, AGAP010731; CLIPA9, AGAP010968; CLIPA12, AGAP011781; CLIPA13, AGAP011783; CLIPA14, AGAP011788; CLIPB2, AGAP003246; CLIPB3, AGAP003249; CLIPB4, AGAP003250; CLIPB8, AGAP003057; CLIPB9, AGAP013442; CLIPB10, AGAP003058; CLIPB13, AGAP004855; CLIPB14, AGAP010833; CLIPB15, AGAP009844; CLIPC1, AGAP008835; CLIPC2, AGAP004317; CLIPC3, AGAP004318; CLIPC5, AGAP000571; CLIPC6, AGAP000315; CLIPC9, AGAP004719; CLIPC10, AGAP000572; CLIPD4, AGAP002811; CLIPD6, AGAP002813; CLIPD7, AGAP008998; CLIPD8, AGAP002784; CLIPE2, AGAP011782; CLIPE4, AGAP010530; CLIPE5, AGAP010547; CLIPE6, AGAP011785; CLIPE7, AGAP011786; PPO6, AGAP004977; CTL4, AGAP005335; SRPN3, AGAP006910.

## Supporting Information

Figure S1 **SPCLIP1 genomic organization, multiple sequence alignment, and phylogenetic analysis.** (**A**) In the top diagram genes are indicated above and below a 55 kb region of *A. gambiae* chromosome 3L depending on whether they are encoded by the positive or negative DNA strand, respectively. The bottom diagram shows an expanded view of an 8.2 kb region indicated in red in the top diagram to illustrate the experimentally derived intron-exon boundaries of *SPCLIP1* and its tail-to-tail orientation with *CLIPA7*. Coding regions are depicted with dark gray boxes, untranslated regions with white boxes, and introns with black lines. Features within both diagrams are drawn to scale. The *SPCLIP1* gene does not correspond to any gene model in the *A. gambiae* genome annotation and is only present as a SNAP prediction. (**B**) Alignment of SPCLIP1 with representative members of the CLIP subfamilies A–D. The N-terminal CLIP domain is indicated by a blue background. Shaded residues indicate consensus similarity, light gray; consensus identity, dark gray; conserved cysteine, yellow; CLIPA and SPCLIP1 conserved tyrosine, red. Stars indicate the positions of the catalytic triad residues and lines connect cysteines involved intramolecular disulfide bonds. The black outline indicates the predicted activation cleavage position in the CLIPB, C and D zymogens. (**C**) Unrooted tree generated from analysis of the protease domain of 35 members of the *A. gambiae* CLIP family. Colored regions highlight the major subfamilies: CLIPA, yellow; CLIPB, blue; CLIPC, green; CLIPD, orange. White circles indicate bootstrap values >80.
(TIF)

Figure S2 **TEP1 is required for CLIPA8 and PPO activation.** (**A**) Reducing western analysis of CLIPA8 in hemolymph collected from control ds*LacZ* injected and *TEP1* and *CLIPA8* kd mosquitoes after injection with *E. coli* bioparticles. CLIPA8-C indicates the CLIPA8 cleavage product which is markedly reduced in TEP1 silenced mosquitoes. Blot was re-probed with an antibody against SRPN3 to confirm equal loading. (**B**) PO activity measured in hemolymph samples collected from ds*TEP1*, ds*CLIPA8* and control ds*LacZ* treated mosquitoes 6 h after injection with bacteria.
(TIF)
