# Supplementary figures and images for: The CLIP-Domain Serine Protease Homolog SPCLIP1 Regulates Complement Recruitment to Microbial Surfaces in the Malaria Mosquito Anopheles gambiae
Source: PLoS Pathog. 2013 Sep 5;9(9):e1003623. doi: 10.1371/journal.ppat.1003623 (PMC3764210; doi:10.1371/journal.ppat.1003623)

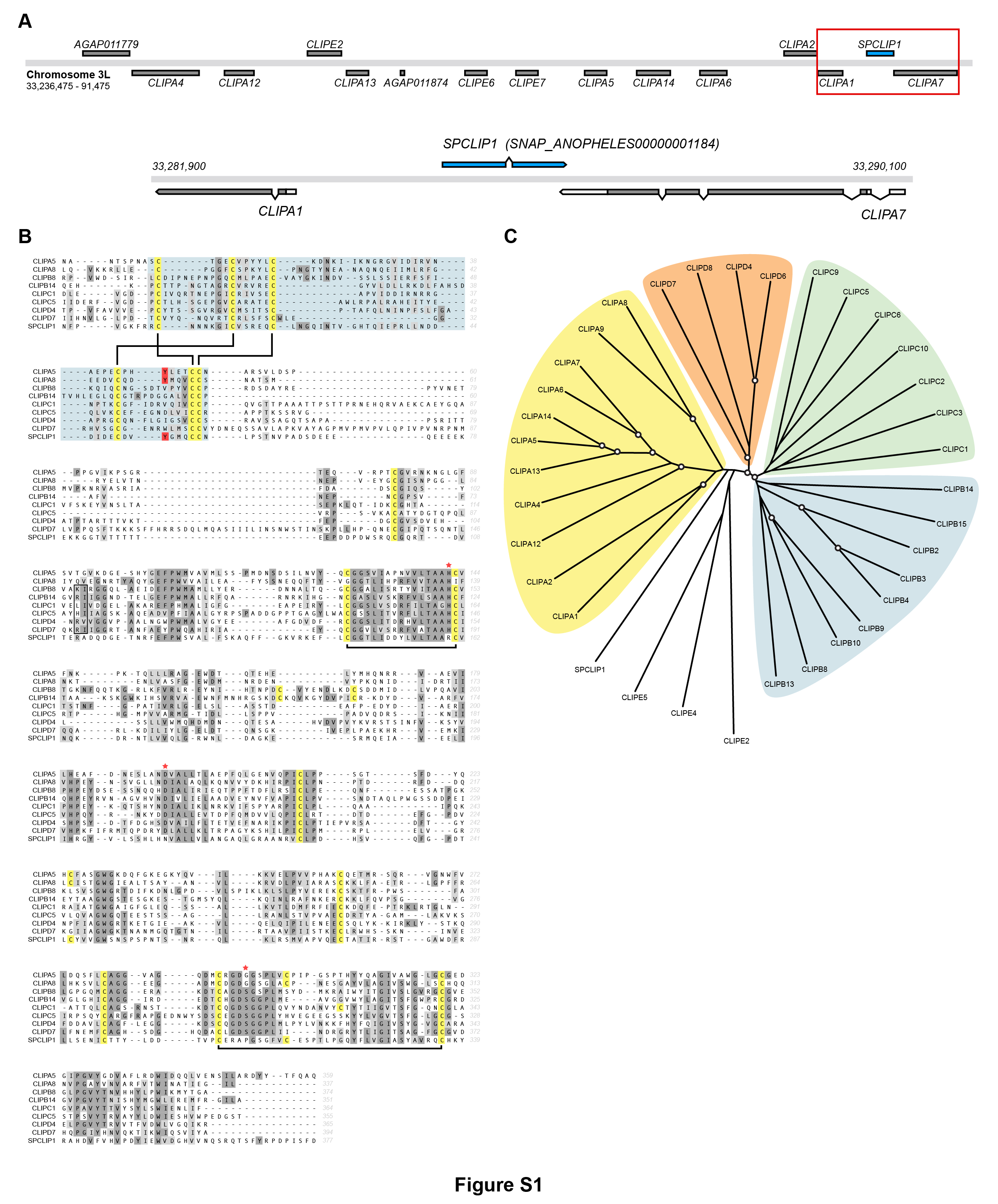

Supplement: Figure S1 — SPCLIP1 genomic organization, multiple sequence alignment, and phylogenetic analysis. (A) In the top diagram genes are indicated above and below a 55 kb region of A. gambiae chromosome 3L depending on whether they are encoded by the positive or negative DNA strand, respectively. The bottom diagram shows an expanded view of an 8.2 kb region indicated in red in the top diagram to illustrate the experimentally derived intron-exon boundaries of SPCLIP1 and its tail-to-tail orientation with CLIPA7. Coding regions are depicted with dark gray boxes, untranslated regions with white boxes, and introns with black lines. Features within both diagrams are drawn to scale. The SPCLIP1 gene does not correspond to any gene model in the A. gambiae genome annotation and is only present as a SNAP prediction. (B) Alignment of SPCLIP1 with representative members of the CLIP subfamilies A–D. The N-terminal CLIP domain is indicated by a blue background. Shaded residues indicate consensus similarity, light gray; consensus identity, dark gray; conserved cysteine, yellow; CLIPA and SPCLIP1 conserved tyrosine, red. Stars indicate the positions of the catalytic triad residues and lines connect cysteines involved intramolecular disulfide bonds. The black outline indicates the predicted activation cleavage position in the CLIPB, C and D zymogens. (C) Unrooted tree generated from analysis of the protease domain of 35 members of the A. gambiae CLIP family. Colored regions highlight the major subfamilies: CLIPA, yellow; CLIPB, blue; CLIPC, green; CLIPD, orange. White circles indicate bootstrap values >80. (TIF) [file ppat.1003623.s001.tif]

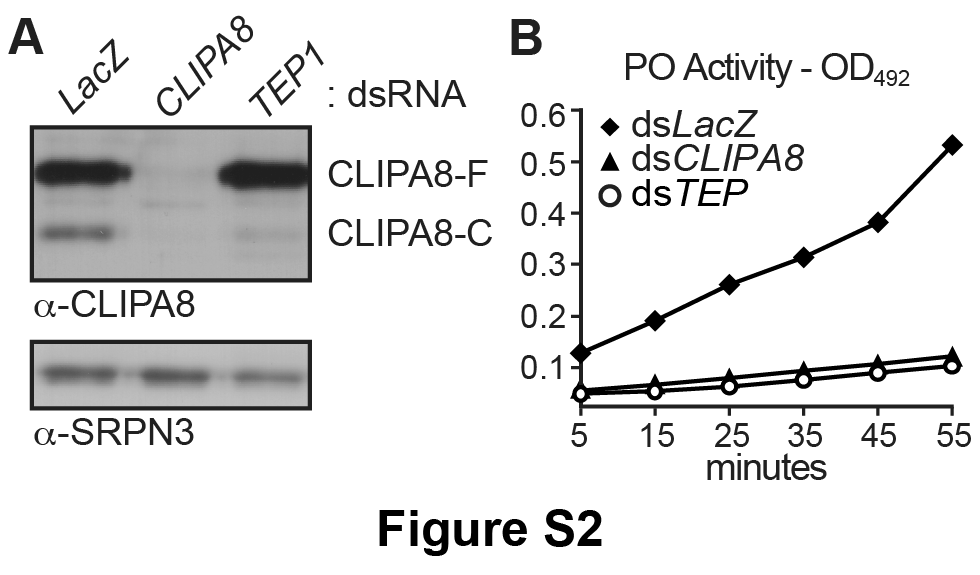

Supplement: Figure S2 — TEP1 is required for CLIPA8 and PPO activation. (A) Reducing western analysis of CLIPA8 in hemolymph collected from control dsLacZ injected and TEP1 and CLIPA8 kd mosquitoes after injection with E. coli bioparticles. CLIPA8-C indicates the CLIPA8 cleavage product which is markedly reduced in TEP1 silenced mosquitoes. Blot was re-probed with an antibody against SRPN3 to confirm equal loading. (B) PO activity measured in hemolymph samples collected from dsTEP1, dsCLIPA8 and control dsLacZ treated mosquitoes 6 h after injection with bacteria. (TIF) [file ppat.1003623.s002.tif]
